# Proinflammatory genotype is associated with the frailty phenotype in the English Longitudinal Study of Ageing

**DOI:** 10.1007/s40520-015-0419-z

**Published:** 2015-08-07

**Authors:** Krisztina Mekli, James Y. Nazroo, Alan D. Marshall, Meena Kumari, Neil Pendleton

**Affiliations:** Cathie Marsh Institute for Social Research, School of Social Sciences, University of Manchester, Humanities Bridgeford Street, Oxford Road, Manchester, M13 9PL UK; Department of Epidemiology and Public Health, University College London, London, UK; Clinical and Cognitive Neurosciences, Institute of Brain, Behaviour and Mental Health, University of Manchester, Manchester, UK; Institute for Social and Economic Research, University of Essex, Essex, UK

**Keywords:** Frailty, SNP, TNF, Biomarker

## Abstract

**Background:**

Frailty is a state of increased vulnerability to poor resolution of homeostasis after a stressor event, which increases the risk of adverse outcomes including falls, disability and death. The underlying pathophysiological pathways of frailty are not known but the hypothalamic–pituitary–adrenal axis and heightened chronic systemic inflammation appear to be major contributors.

**Methods:**

We used the English Longitudinal Study of Ageing dataset of 3160 individuals over the age of 50 and assessed their frailty status according to the Fried-criteria. We selected single nucleotide polymorphisms in genes involved in the steroid hormone or inflammatory pathways and performed linear association analysis using age and sex as covariates. To support the biological plausibility of any genetic associations, we selected biomarker levels for further analyses to act as potential endophenotypes of our chosen genetic loci.

**Results:**

The strongest association with frailty was observed in the Tumor Necrosis Factor (*TNF*) (rs1800629, *P* = 0.001198, *β* = 0.0894) and the Protein Tyrosine Phosphatase, Receptor type, J (*PTPRJ*) (rs1566729, *P* = 0.001372, *β* = 0.09397) genes. Rs1800629 was significantly associated with decreased levels of high-density lipoprotein (HDL) (*P* = 0.00949) and cholesterol levels (*P* = 0.00315), whereas rs1566729 was associated with increased levels of HDL (*P* = 0.01943). After correcting for multiple testing none of the associations remained significant.

**Conclusions:**

We provide potential evidence for the involvement of a multifunctional proinflammatory cytokine gene (*TNF*) in the frailty phenotype. The implication of this gene is further supported by association with the endophenotype biomarker results.

**Electronic supplementary material:**

The online version of this article (doi:10.1007/s40520-015-0419-z) contains supplementary material, which is available to authorized users.

## Introduction

Frailty refers a state of increased vulnerability to poor resolution of homeostasis after a stressor event, which increases the risk of adverse outcomes, including falls, delirium and disability [1]. The mechanistic pathophysiological pathways of frailty are not known, but there is evidence for the involvement of steroid hormones and the immune system [2, 3]. During ageing, physiological decline occurs in steroid hormone levels, as observed in males where free testosterone declines at the rate of 1 % per year [4]. Similarly, in females cycling estradiol production is replaced by very low, constant, estradiol levels after the age of about 50 [5]. In both sexes, dehydroepiandrosterone (DHEA) and its sulphated derivate (DHEAS) levels gradually decline in later life due to the decreased activity of adrenal glands [6]. These hormones are known to effect changes in proinflammatory cytokine levels, for example, testosterone was shown to suppress interleukin-6 (IL-6) mRNA levels in osteoblastic cell line [7] and reduce the expression and secretion of tumor necrosis factor (TNF) in macrophages [8]. Estradiol in human bone-derived cells inhibits TNF-induced IL-6 production [9]. Finally, DHEA concentration has been shown to inhibit IL-6 secretion from peripheral blood mononuclear cells [10]. The increased levels of proinflammatory cytokines (IL-1, IL-6 and TNF) and acute-phase proteins, such as C-reactive protein (CRP), results in a chronic, low-grade systemic inflammatory state of the ageing immune system, termed “inflamm-ageing”. This chronic inflammation leads to long-term tissue damage and is detrimental for longevity [11]. Increased levels of CRP and IL-6 have been associated with increased mortality in the elderly [12, 13]. Studies have shown a large body of evidence suggestive of a heightened inflammatory state in frail older individuals, as marked by increase of these inflammatory markers compared with levels observed in robust older controls [14–16].

There have been only a few studies on the genetic determinants of frailty and those that have been conducted have somewhat conflicting results. A candidate gene study found that two single nucleotide polymorphs (SNP) of the *IL*-*6* gene were weakly associated with frailty but not with serum IL-6 levels [17], whilst another showed that a SNP in the *CRP* gene (rs1205) is associated with frailty scores, albeit counter intuitively with presence of frailty related to lower levels of CRP [18]. The largest study so far (in terms of number of SNPs investigated) has found weak associations between frailty and genetic variants of genes involved in pathways related to apoptosis and metabolism of amino acids in a sample of elderly women [19]. However, these results did not survive correction for multiple testing.

In this study, we conducted a candidate gene association study using a standardized frailty phenotype [20] in 3160 community dwelling individuals over the age of 50 in the English Longitudinal Study of Ageing (ELSA) cohort. We selected genes involved in the steroid hormone biosynthesis and inflammation pathways, as evidence in the literature indicates their possible involvement in frailty pathophysiology (for example, DHEAS [21], IL-6, TNF and C-reactive protein [14]). We also selected genetic variants of key genes of the steroid hormone and inflammatory pathways, such as *HSD11B1*, *SULT1E1*, *SULT2A1*, *IL1B* and *IFNG*. We hypothesized that SNPs within these genes, especially those ones which have been shown to affect the expression of their genes such as rs1800795 for *IL6* [22] and rs1800629 for *TNF* [23] or have shown associations with metabolite levels such as rs2547231 and rs182420 for *SULT2A1* [24], will show significant association to frailty phenotype.

To support the biological plausibility of any genetic association with the frailty phenotype, we also conducted association analyses with selected biomarkers that act as potential endophenotypes for our chosen genetic loci: high-sensitivity C-reactive protein (hsCRP) as inflammatory marker, high-density lipoprotein (HDL), total cholesterol and DHEAS as markers for steroid metabolism.

## Materials and methods

### Participants

The analyses are performed on a sample of 3160 participants drawn from Wave 2 (2004) and Wave 4 (2008) of the English Longitudinal Study of Ageing (ELSA). Detailed description of ELSA can be found elsewhere [25], (http://www.ifs.org.uk/ELSA). Briefly ELSA is prospective cohort study representative of older men and women living in England who originally participated in the Health Survey for England in 1998, 1999 or 2001 (http://www.natcen.ac.uk/series/health-survey-for-england). The participants have been participated biannually in a computer-assisted personal interview (Core dataset) and every 4 years for a separate nurse assessment (Nurse dataset), carried out shortly after the interview. During the nurse assessment participants gave blood for genetic and biomarker analysis.

### Genetic and biomarker data

Genotype data of 620 Single Nucleotide Polymorphisms (SNP) for 3160 participants were obtained from a publicly available ELSA DNA Repository (EDNAR).

Genotyping was performed by Illumina (San Diego, CA) as part of a 1536 Goldengate custom SNP panel using high-throughput BeadArrayTM technology.

For the comparative endophenotype analysis, biomarker data were drawn from both the Wave 2 (2004) and Wave 4 (2008) Nurse dataset. We used the following analytes from W2: blood total cholesterol (mmol/l), HDL (mmol/l), and hsCRP (mg/l). HsCRP >10 mg/l results were excluded from the analysis as they indicate ongoing acute-phase response [26]. From W4, we used the DHEAS level (μmol/l). Blood samples were analysed at the Royal Victoria Infirmary laboratory in Newcastle upon Tyne, UK (detailed description of blood analyses can be found in [27]).

### Phenotypic measures

Frailty status assessment was based on the 5-item criteria by Fried and colleagues [20], derived for both Wave 2 and Wave 4. This is a widely used measure which determines the condition based on specific criteria, such as unintentional weight loss, exhaustion, low physical activity, slowness and weakness. As with previous studies using Frailty Phenotype (FP) criteria, we conducted a study specific operationalization in ELSA, as follows. Weight loss (item 1) was defined as present if changes in measured weight loss between waves were equal or greater than 8 % baseline values. Exhaustion (item 2) was recorded present if a positive answer to both of the questions from the Center for Epidemiological Studies depression questionnaire (CES-D) [28]: whether respondent felt everything they did during the past week was an effort and whether respondent could not get going much of the time during the past week.

Low physical activity (item 3) used self-report questions on work activity and recreational physical activity. In short, if subject reported they did not work and performed only mild sport activity not more than once a week, they were considered positive for this item.

Respondent is considered positive for slowness (item 4) if the average of the two timed 8 feet (=240 cm) walk test falls into the slowest 20 % of population, adjusting for sex and height.

Respondent was assessed as frail for weakness (item 5) if the average of the 3 dominant hand grip strength (GS) measures by handheld dynamometer falls into lowest 20 % of the population stratified by sex and body mass index (BMI). The latter 2 items were assessed in the whole ELSA wave 2 and wave 4 cohorts with available data then the cut-off points were applied for the genotyped sub-cohort. (Detailed description of variables used for frailty assessment can be found in the online Supplementary material Appendix 1.)

For analysis purposes, we used these variables both as a summed count of the number of frailty items present (0–5) and as an ordinal frailty status measure (frail if they possessed 3–5 items, pre-frail if 1–2 items and robust if none). Biomarker data were treated as continuous variables and were normally distributed. However, hsCRP and DHEAS data were skewed; therefore a square root transformation was performed for genetic association analysis purposes to normalize the distribution.

### Statistical analysis

We used the Plink software for genetic association analyses [29]. Associations between genotypes and number of frailty items present (0–5) and between genotypes and biomarker levels, and were tested with linear regression using sex and age as covariates. Results of the most significant associations were re-tested by ordered logistic regression using frailty status as dependent variable within the Stata12 software (Stata Corporation, http://www.stata.com/). Stata12 was also used for demographic, phenotypic and biomarker analysis. Linkage disequilibrium blocks in *PTPRJ* gene were generated and visualized with HaploView software [30]. Genetic power calculation was performed by Quanto software (http://biostats.usc.edu/Quanto.html).

## Results

### Demographic, phenotypic and biomarker distributions

Tables 1 and 2 show the demographic and frailty phenotype results for the participants,, respectively. There were more females than males in the sample with no significant differences between the mean ages. There is evidence of a relationship between sex and the FP but this did not achieve statistical significance at the 5 % level in Wave 2 (*P* = 0.068) and was not significant in Wave 4 (*P* = 0.106) (Fischer’s exact test).

**Table 1:** Demographic result of participants

	Total sample	Males	Females
Number of participants	3160	1466	1694
Mean age years (SE) in Wave 2	68.3 (0.10)	68.27 (0.14)	68.32 (0.14)
Mean age years (SE) in Wave 4	72.05 (0.11)	71.93 (0.17)	72.15 (0.15)

*SE* standard error of the mean

**Table 2:** Frailty status results of participants in Wave 2 and Wave 4

		Frailty categories					
		Robust	Pre-frail		Frail		
	Mean number of items (SD)	0 item	1 item	2 items	3 items	4 items	5 items
		Number (%)	Number (%)	Number (%)	Number (%)	Number (%)	Number (%)
Total sample							
Wave 2	0.52 (0.83)	1745 (63.55)	694 (25.27)	212 (7.72)	67 (2.44)	22 (0.80)	6 (0.22)
Wave 4	0.65 (0.91)	1148 (56.97)	546 (27.10)	225 (11.17)	66 (3.28)	28 (1.39)	2 (0.10)
Males							
Wave 2	0.5 (0.78)	817 (64.03)	330 (25.86)	88 (6.90)	34 (2.66)	7 (0.55)	0 (0)
Wave 4	0.61 (0.86)	540 (58.89)	241 (26.28)	100 (10.91)	29 (3.16)	6 (0.65)	1 (0.11)
Females							
Wave 2	0.54 (0.87)	928 (63.13)	364 (24.76)	124 (8.44)	33 (2.24)	15 (1.02)	6 (0.41)
Wave 4	0.69 (0.95)	608 (55.37)	305 (27.78)	125 (11.38)	37 (3.37)	22 (2.0)	1 (0.09)

*SD* standard deviation

Table 3 shows the mean biomarker levels for the entire population and for the two sexes. All the biomarker levels were significantly different between sexes on the *P* < 0.05 level.

**Table 3:** Biomarker level results

	Total sample	Males	Females
Cholesterol (mmol/l) (SE)	5.89 (0.022)	5.55 (0.030)	6.17 (0.030)
HDL (mmol/l) (SE)	1.52 (0.007)	1.38 (0.009)	1.64 (0.009)
hsCRP (max 10) (mg/l) (SE)	2.58 (0.040)	2.48 (0.059)	2.67 (0.056)
DHEAS (µmol/l) (SE)	1.9 (0.034)	2.38 (0.059)	1.53 (0.033)

All biomarker levels were significantly different in the sexes (two-group mean-comparison *t* test, *P* < 0.05 level)

Analyses of hsCRP and DHEAS were done with square root-transformed values

*HDL* high-density lipoprotein, *hsCRP* high-sensitivity C-reactive protein, *DHEAS* dehydroepiandrosterone-sulphate, *SE* standard error of the mean

To explore the predictive value of these biomarkers, we conducted a Spearman’s correlation test. The analysis revealed that all biomarkers were significantly correlated with the number of frailty items at least on *P* < 0.005 level (HDL, Spearman’s rho = −0.054) or below the *P* < 0.0001 level (cholesterol, hsCRP and DHEAS, Sperman’s rho: −0.118, 0.098 and −0.143, respectively). Table 4 shows the mean biomarker levels, according to frailty categories (robust, pre-frail and frail).

**Table 4:** Biomarker levels according to frailty categories

	Cholesterol (mmol/l) (SE)	HDL (mmol/l) (SE)	hsCRP (max 10) (mg/l) (SE)	DHEAS (µmol/l) (SE)
Robust	5.978015 (0.029)	1.536526 (0.009)	2.385388 (0.050)	2.034286 (0.043)
Pre-frail	5.757667 (0.040)	1.502333 (0.013)	2.772649 (0.080)	1.678685 (0.060)
Frail	5.374468 (0.146)	1.451064 (0.040)	3.047945 (0.278)	1.8875 (0.263)

*HDL* high-density lipoprotein, *hsCRP* high-sensitivity C-reactive protein, *DHEAS* dehydroepiandrosterone-sulphate, *SE* standard error of the mean

### Genetic association results

Of the available 620 SNPs, 15 were out of Hardy–Weinberg equilibrium (*P* < 0.01), and a further 15 had a minor allele frequency (MAF) below 5 %, so these were excluded from the genetic association analysis, resulting in 590 SNPs used.

**Fig. 1:**
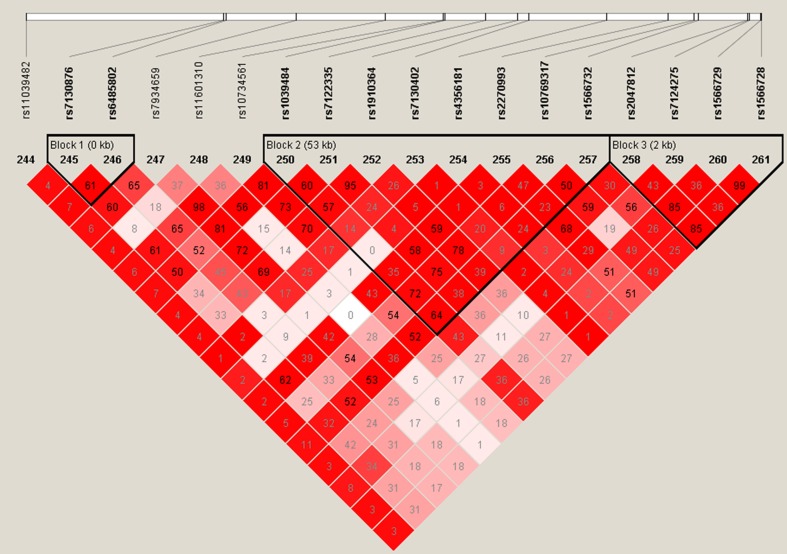
Linkage disequilibrium (LD) blocks within the *PTPRJ* gene. Rs2047812, rs1566729 and rs1566728 are in strong correlation with each other (*R* ^2^ ≥ 0.85), indicating that the observed associations are possibly originated from the same signal

Table 5 shows the most significant results of the genetic association analysis (for results for each of the 590 SNPs see online Supplementary material Appendix 2). The SNP which was most significantly associated with the number of frailty items (0–5) phenotype is rs1800629 (*β* = 0.0894, *P* = 0.001198) in the promoter region of Tumour Necrosis Factor gene (*TNF*) on Chromosome 6. Four other SNPs (rs1566729, rs1566728, rs2047812 and rs611646) were associated below the *P* = 0.01 level (*β* values 0.09397, 0.09043, 0.08481 and −0.06407, respectively). The first three of these are located within the Protein Tyrosine Phosphatase, Receptor type, J (*PTPRJ*) gene on chromosome 11. Two of these SNPs, rs1566729 and rs1566728, are in very high linkage disequilibrium (LD) with each other (*r*^2^ = 0.99) and rs2047812 with both of them (*r*^2^ = 0.85) thus they probably indicate the same signal (Fig. 1). Finally, the fourth SNP, rs611646 which appeared to reduce the number of frailty items (*β* = −0.06407, *P* = 0.003904), is an intron variant within the Ataxia Telangiectasia Mutated (*ATM*) gene on chromosome 11.

**Table 5:** Most significant results of the genetic association analysis

SNP	Minor Allele	Linear regression										Ordered logistic regression	
		Number of frailty items		HDL (mmol/l)		Cholesterol (mmol/l)		hsCRP max 10 (mg/l) (square root)		DHEAS (µmol/l) (square root)		Frailty status	
		*β*	*P*	*β*	*P*	*β*	*P*	*β*	*P*	*β*	*P*	Coefficient	*P*
Rs1800629	A	0.0894	0.001198	−0.03022	0.009489	−0.11	0.003151	−0.005621	0.7921	0.001283	0.9422	0.2346339	0.001
Rs1566729	A	0.09397	0.001372	0.02898	0.01943	0.02571	0.5168	−0.002585	0.9087	0.03404	0.07158	0.2088451	0.006
Rs1566728	G	0.09043	0.002069	0.02947	0.01736	0.02655	0.5035	−0.004463	0.8431	0.03435	0.06942	0.1994975	0.008
Rs2047812	A	0.08481	0.002451	0.03593	0.002294	0.03172	0.4002	0.003949	0.8536	0.03505	0.0527	0.2094985	0.004
Rs611646	T	−0.06407	0.003904	−0.00777	0.4067	0.0001194	0.9968	−0.01392	0.4138	−0.00173	0.9042	−0.127991	0.027
Rs4646316	A	0.06199	0.01552	0.01809	0.09439	0.05547	0.1083	0.02149	0.2726	0.00997	0.5432	0.1695163	0.01

*HDL* high-density lipoprotein, *hsCRP* high-sensitivity C-reactive protein, *DHEAS* dehydroepiandrosterone-sulphate

None of these results survives the Bonferroni-correction (*P* < 8.474E–05 for 590 tests).

Retesting the nominally significant results (*P* < 0.05) of the genetic association analysis with ordered logistic regression using frailty status (robust, pre-frail and frail) phenotype confirmed the previous results. The most significantly associated variant remained rs1800629 of *TNF* (coefficient = 0.2346, *P* = 0.001). Of the *PTPRJ* genetic variants, the same three SNPs were significantly associated with this phenotype below the *P* = 0.01 threshold (rs2047812, coefficient = 0.2095, *P* = 0.004; rs1566729, coefficient = 0.2088, *P* = 0.006 and rs1566728, coefficient = 0.1995, *P* = 0.008). Finally, another SNP reached the *P* = 0.01 significance level, rs4646316 (coefficient = 0.1695, *P* = 0.01) an intronic variant within the Catechol-*O*-methyltransferase (*COMT*) gene on chromosome 22.

To provide support for biological plausibility for genetic findings, we selected steroid hormone biosynthesis and inflammatory pathway-related biomarkers as endophenotypes and examined if any of the SNPs were significantly associated with their levels.

TNF SNP rs1800629 was significantly associated with decreased cholesterol level (*β* = −0.11, *P* = 0.003151) and decreased HDL level (*β* = −0.03022, *P* = 0.009489). Of the 3 *PTPRJ* SNPs each was significantly associated with increased HDL levels (*β* values 0.02898–0.03593, *P* values 0.002294–0.01943), and at least on a suggestive level with increased square root levels of DHEAS (*β* values 0.03404–0.03505, *P* values 0.0527–0.07158). Finally, rs611646 or rs4646316 did not have an effect on any of the biomarker levels.

Finally, we included HDL, cholesterol and hsCRP levels separately in a multivariate model to test if adding these biomarker levels attenuates the association between SNPs and frailty status. Including hsCRP in the model reduced the coefficient to 0.1776 for rs1566728 and to 0.1898 for rs2047812, whereas cholesterol and HDL in the model affected the coefficient for rs611646 (−0.118 and −0.116, respectively) (Table 6).

**Table 6:** Results of the multivariate model

SNP	Cholesterol levels in the model		HDL levels in the model		Square root hsCRP levels in the model	
	Coefficient	*P*	Coefficient	*P*	Coefficient	*P*
Rs1800629	0.22144	0.002	0.229437	0.001	0.238789	0.002
Rs1566729	0.213732	0.005	0.219683	0.004	0.185953	0.021
Rs1566728	0.204545	0.007	0.210509	0.006	0.177571	0.028
Rs2047812	0.214303	0.003	0.222936	0.002	0.189815	0.013
Rs611646	−0.117873	0.043	−0.115613	0.047	−0.135985	0.027
Rs4646316	0.180721	0.006	0.177258	0.007	0.190133	0.006

*HDL* high-density lipoprotein, *hsCRP* high-sensitivity C-reactive protein

## Discussion

In this study, we investigated the association between genes involved in steroid hormone metabolism and inflammatory pathways and the frailty phenotype. Our results show evidence of the involvement of *TNF*, *PPRJ*, *ATM* and *COMT* genes.

Our most significant genetic finding is with the *TNF* locus on chromosome 6. The *TNF* gene encodes a proinflammatory cytokine, mainly secreted by macrophages. This cytokine is involved in the regulation of a wide spectrum of biological processes including cell proliferation, differentiation, apoptosis, lipid metabolism, and coagulation and has been implicated in a variety of diseases, such as autoimmune diseases, insulin resistance, and cancer [31]. The most significant SNP, rs1800629 is located in the promoter region of the *TNF* gene causing a G > A transition. Functional studies suggest that the rare A allele is a much more powerful transcriptional activator than the common G allele [23]. In our study, the rare A allele was significantly associated with possession of increasing numbers of frailty items and becoming frail. This agrees with the observations of elevated TNF levels in frail individuals [15]. This SNP was significantly associated with decreased HDL and cholesterol levels as well in our study. Relationships between TNF and blood lipid levels have been investigated before. During septic shock increased TNF levels were found to be accompanied by a significant decline in total serum cholesterol [32], whilst TNF inhibitor treatment was associated with increased levels of HDL and total cholesterol in rheumatoid arthritis patients [33]. Studies of elderly participants found association between lower blood lipid levels and survival. For example, a 2-year follow-up study of very old and frail individuals has found that HDL cholesterol levels were significantly lower among those who died compared with survivors [34], whilst in another 3-year follow-up study subjects with low total cholesterol levels were at a higher risk of dying [35]. Finally, in a genome-wide association (GWA) study of over 100,000 individuals of European ancestry rs1800629 A allele was associated with lower cholesterol (*P* = 6.81E−09) and triglyceride (*P* = 7.90E−09) levels [36]. Despite these findings, it is possible that the frailty increasing and cholesterol and HDL-level decreasing effects of TNF are manifested through different pathways.

The role of the immune system in FP was not corroborated by other genes, such as *CRP*, *IL6* or *IL6R* in our study. It may indicate that the effect of TNF manifests though other pathways rather than the inflammatory, such as apoptosis.

Our second most significant genetic association hit was with SNP rs1566729 which lies within the *PTPRJ* gene. *PTPRJ* is a member of the protein tyrosine phosphatase (PTP) family. PTPs are known to be signalling molecules that regulate a variety of cellular processes, including cell growth, differentiation, mitotic cycle and cancer-associated signalling processes. *PTPRJ* has been implicated in breast, colon, lung and thyroid cancers [37], and it is expressed by all resting leukocytes and upregulated following in vitro stimulation, thus it appears to be a T cell activating molecule [38]. A recent study suggests a possible connection between *TNF* and *PTPRJ*. In a mouse melanoma model treatment with TNF yielded the downregulation of *PTPRJ* [39]. The 3 *PTPRJ* SNPs were investigated by Teslovich and colleagues [36] who reported an increase in HDL levels (P values range between 1.77E−08: rs1566729 to 2.71E−08: rs1566728), as we did, but this contradicts the literature showing a negative association with frailty that we have just described [34]. A further contradiction is that these SNPs appear to be associated with increased DHEAS levels, which is thought to be protective against frailty [40]. A possible interpretation of this counter-intuitive finding may be that an effect of PTPRJ antagonises the HDL, DHEAS level-increasing effects and increases frailty.

The third gene below the 0.01 significance threshold is *ATM*. ATM protein is a high-molecular-weight PI3K-family kinase. It plays many important cytoplasmic roles where it phosphorylates hundreds of protein substrates that activate and coordinate cell-signalling pathways involved in cell-cycle checkpoints, nuclear localization, gene transcription and expression, the response to oxidative stress, apoptosis and nonsense-mediated decay, among others [41]. With this wide-range of functions in the cell, this gene and its protein product are plausible candidates for frailty. Our top SNP within this gene, rs611646 has been associated with breast cancer in interaction with *BRCA1* in a study of Chinese Han subjects [42] indicating its possible functionality.

The ordered logistic regression showed the possible role of a fourth gene, *COMT*. The protein product of this gene catalyses the transfer of a methyl group to catecholamines, including neurotransmitters dopamine, epinephrine and norepinephrine, but it also acts on oestrogen, a member of the steroid biosynthesis pathway [43]. Rs4646316 within this gene has been shown to be associated with schizophrenia [44] but not with any biomarker level in our study, making this result difficult to interpret.

Including the biomarkers into the model only slightly attenuated the relationship between frailty status and the SNPs in PTPRJ and ATM genes. It may indicate that the relationship between these variables is not a direct mechanistic one or that biomarker levels and SNP variants are independently associated to frailty.

The measured biomarkers were all significant predictors for the number of frailty items possessed, but their individual effects were small. This is in agreement with the genetic findings which indicate the involvement of several pathways, each with limited effects.

This study has a number of limitations. First, only a proportion of the ELSA sample were genotyped and the prevalence of frailty in this group was slightly lower than in the full sample, thus it may not be representative for the whole United Kingdom population of older adults. Second, the frailty phenotype is constructed using study specific items from the extensive ELSA public archive, although as in many published studies in we used an approach based on the method published by Fried and colleagues [20]. Finally, the analysed biomarkers, genes and genotypic variants were selected from a publicly available dataset rather than specifically for the purpose of this study. However, this did include SNPs and biomarkers relevant to the pathways we set out to investigate.

The strength of our study is the adequate sample size. The effective sample size required to test whether a gene main effect is associated with a continuous trait for 99 % power, *α* = 0.05 (two-sided test), assuming outcomes that are modelled using linear regression (mean 0.52, SD 0.83, genetic effect = 0.01, MAF = 0.05) is 1828, which is given in our study. Despite this, it would advantageous to replicate our findings. Another advantage is the use of biomarker levels as endophenotypes, which offers additional biological plausibility for our findings.

In conclusion, our study provides evidence for the involvement of the TNF in the pathophysiology of frailty in line with previous observations in the literature. We also report new observations of the involvement of a protein tyrosine phosphatase (*PTPRJ*), Ataxia Telangiectasia Mutated (*ATM*) and Catechol-*O*-methyltransferase (*COMT*) in the frailty phenotype. Although we use biomarker endophenotypes to support plausibility of these genetic associations, replication in other cohorts is encouraged. Nevertheless, these findings provide opportunities to better understand the biological processes underpinning frailty, which may over time lead to possible interventions.

## Electronic supplementary material

Below is the link to the electronic supplementary material.

Appendix 1 Variables used in the English Longitudinal Study of Ageing to operationalize the Frailty Phenotype (DOCX 16 kb)

Appendix 2 Results of the genetic association analysis for the 590 SNPs analysed (XLSX 95 kb)
