# Supplementary material for: Proinflammatory genotype is associated with the frailty phenotype in the English Longitudinal Study of Ageing
Source: Aging Clin Exp Res. 2015 Aug 7;28:413–21. doi: 10.1007/s40520-015-0419-z (PMC4877432; doi:10.1007/s40520-015-0419-z)
Supplement: Supplementary file 1 — Appendix 1 Variables used in the English Longitudinal Study of Ageing to operationalize the Frailty Phenotype (DOCX 16 kb) [file 40520_2015_419_MOESM1_ESM.docx]

**Article title:** Proinflammatory genotype is associated with the frailty phenotype in the English Longitudinal Study of Ageing

**Journal:** Aging Clinical and Experimental Research

**Authors:** Krisztina Mekli, James Y. Nazroo, Alan D. Marshall, Meena Kumari, Neil Pendleton

**Corresponding author**: Krisztina Mekli, Cathie Marsh Institute for Social Research, School of Social Sciences, Humanities Bridgeford Street, Oxford Road, University of Manchester, M13 9PL, United Kingdom. E-mail: [Krisztina.Mekli@manchester.ac.uk](mailto:Krisztina.Mekli@manchester.ac.uk).

**Appendix A**

Variables in the ELSA dataset used to operationalize frailty in Wave 2 and W ave 4

Item 1

We used Wtval (=valid weight [kg], including estimated >130 kg) or Weight (=weight [kg] including unreliable measurements, if the former was not available) to assess this item. The initial bodyweight data was taken from the Health Survey for England dataset (years 1998-2001, Wave 0).

Item 2

We used PScedB (=Whether respondent felt everything they did during the past week was an effort, possible answers: yes/no) and PScedH (=Whether respondent could not get going much of the time during the past week, possible answers: yes/no).

Item 3

We used Wpjact (=Level of physical activity in main job, possible answers: Not applicable/ Sedentary occupation/ Standing occupation/ Physical work- This involves some physical effort/ Heavy manual work-This involves vigorous physical activity), HeActA(=Frequency does vigorous sports or activities, possible answers: more than once a week/ once a week/ one to three times a month/ hardly ever, or never), HeActB (=Frequency does moderate sports or activities, possible answers: more than once a week/ once a week/ one to three times a month/ hardly ever, or never) and HeActC (=Frequency does mild sports or activities, possible answers: more than once a week/ once a week/ one to three times a month/ hardly ever, or never) variables.

Item 4

Variables used for this item are MMWlkA /B (=Time taken for first/second walk 8 feet [seconds]).

Respondent is positive for this item if the average of the two timed walk test falls into the slowest 20% of the wave 2/wave 4 cohort, adjusting for sex (variable Indsex= Definitive sex variable, 1=male, 2=female) and height (variables are Htval= Valid height [cm] or Height= Standing height [cm] including unreliable measurements, if the former was not available).

Moreover, we used MmSchs variable (=Observed mobility status, possible answers: Observed walking without other person's help / using support/ Observed walking with other person's help or using support/ Not observed - in wheelchair/ Not observed - bed bound/ Not observed - uncertain if respondent has impairment) and assessed respondent as frail if this variable indicated that (s)he was wheelchair- or bed-bound.

Item 5

We used the average of 3 variables Mmgsd1/2/3 (= Grip strength: 1^st^/2^nd^/3^rd^ measurement dominant hand [kg]) indicating grip strength measured with a hand-held dynamometer.

Respondent was assessed as frail for this item if the average of the 3 dominant hand grip strength measures falls into lowest 20% of the wave 2/wave 4 cohort stratified by sex (variable as before) and body mass index (BMI, variables: Bmival= Valid BMI - including estimated>130kg [kg/m2] or Bmi= BMI - including unreliable measurements [kg/m2], if the former was not available).

Wtval and Weight variables were measured in Wave 0, Wave 2 and Wave 4, all the others in Wave 2 and Wave 4.
